# Propylene Glycol Poisoning From Excess Whiskey Ingestion: A Case of High Osmolal Gap Metabolic Acidosis

**DOI:** 10.1177/2324709615603722

**Published:** 2015-09-03

**Authors:** Courtney A. Cunningham, Kevin Ku, Gloria R. Sue

**Affiliations:** 1 Santa Clara Valley Medical Center, San Jose, CA, USA; 2 Stanford University School of Medicine, Stanford, CA, USA

**Keywords:** propylene glycol, anion gap, osmolal gap, ingestion, alcohol, intoxication, Food and Drug Administration

## Abstract

In this report, we describe a case of high anion gap metabolic acidosis with a significant osmolal gap attributed to the ingestion of liquor containing propylene glycol. Recently, several reports have characterized severe lactic acidosis occurring in the setting of iatrogenic unintentional overdosing of medications that use propylene glycol as a diluent, including lorazepam and diazepam. To date, no studies have explored potential effects of excess propylene glycol in the setting of alcohol intoxication. Our patient endorsed drinking large volumes of cinnamon flavored whiskey, which was likely Fireball Cinnamon Whisky. To our knowledge, this is the first case of propylene glycol toxicity from an intentional ingestion of liquor containing propylene glycol.

## Introduction

Propylene glycol is a widely used organic compound that is approved by the US Food and Drug Administration for use as a preservative in foods and as a solvent in pharmaceuticals.^1^ Propylene glycol is primarily metabolized into lactate and pyruvate.^2^ The Food and Drug Administration classifies propylene glycol as “generally regarded as safe” and regulates the maximally allowable levels of propylene glycol in a wide variety of products of consumption. Recently, several reports have characterized severe lactic acidosis occurring in the setting of iatrogenic unintentional overdosing of medications that use propylene glycol as a diluent, including lorazepam and diazepam.^3,4^ To date, no studies have explored potential effects of excess propylene glycol in the setting of alcohol intoxication. In this report, we describe a case of high anion gap metabolic acidosis with a significant osmolal gap attributed to the ingestion of liquor containing propylene glycol.

## Case Report

A young African-American man with unknown past medical history was found down and unresponsive at a bus stop. The patient had reportedly presented 1 day prior to an emergency department appearing intoxicated. He refused treatment and subsequently eloped. On initial presentation to our emergency department, his blood pressure was 140/97, pulse was 102 beats per minute, respirations were 21 breaths per minute, oxygen saturation was 100% on room air, temperature was 36.6°C, and weight was 67.2 kg. His physical exam was notable for unresponsiveness, a Glasgow Coma Scale score of 6, pinpoint pupils, excessive frothy oral secretions, tongue biting, jaw clenching, and cool extremities. Our initial concern for this patient was an acute drug overdose with possible seizures. The patient was given 0.4 mg of naloxone and was intubated for airway protection given his low Glasgow Coma Scale score. A urine toxicology screen was negative for amphetamines, barbiturates, benzodiazepines, cocaine, opiates, phencyclidine, and other drugs. Serology was notable for an anion gap of 19 mmol/L, a blood alcohol level of 640 mg/dL, and a measured serum osmolality of 490 mOsm/kg. Serum bicarbonate was 21 mmol/L. After accounting for alcohol, the calculated osmolality was 440 mOsm/kg with an osmolal gap of 50 mOsm/kg. A volatile screen demonstrated a propylene glycol level of 238 µg/mL. After 4 hours of fluid resuscitation, the patient regained consciousness and was subsequently extubated. The patient denied ingestions other than alcohol, and distinctly recalled drinking large quantities of cinnamon-flavored whiskey. The patient’s serum osmolality down-trended to 326 mOsm/kg at 20 hours from initial presentation with the administration of intravenous fluids; ethanol was 139 mg/dL, and propylene glycol was 178 µg/mL at this time. Although an initial lactic acid was not obtained, we noted a downtrend in subsequent lactic acid levels from 6.9 mmol/L at 4 hours after initial presentation to 2.7 mmol/L at 20 hours. His renal function remained stable throughout recovery (creatinine 0.7-0.9 mg/dL). At this point the patient was discharged in stable condition.

## Discussion

In this report, we present an interesting case of metabolic acidosis and a large osmolal gap from the excess ingestion of liquor containing propylene glycol. Our patient endorsed drinking large volumes of cinnamon-flavored whiskey, which was likely Fireball Cinnamon Whisky. To our knowledge, this is the first case of propylene glycol toxicity from an intentional ingestion of liquor containing propylene glycol.

Propylene glycol is an ingredient in the increasingly popular liquor Fireball Cinnamon Whisky. Its propylene glycol content is compliant with the Food and Drug Administration regulations stipulating that propylene glycol is allowable in amounts of up to 5% of the overall content of alcoholic beverages. However, this particular liquor was recalled in October 2014 in some European countries out of concern specifically for unsafe levels of propylene glycol.^5^

The Food and Drug Administration regulates propylene glycol levels in a wide variety of products of consumption. Propylene glycol is a diluent found in many foods, cosmetics, and oral medications. In medicine, propylene glycol is found in intravenous formulations of benzodiazepines such as diazepam and lorazepam. Propylene glycol toxicity has previously been reported following accidental overdoses of diazepam and lorazepam in the inpatient setting.^3,4^ The acute clinical effects of propylene glycol poisoning are primarily central nervous system depression and lactic acidosis.^6^ Other effects include skin/soft tissue necrosis, cardiac dysrhythmias, hypotension, seizure, and hemolysis.^6^

Propylene glycol is primarily metabolized by alcohol dehydrogenase in liver into lactic acid, then pyruvic acid. Propylene glycol is toxic at elevated amounts, which can lead to severe lactic acidosis and renal failure. Fomepizole has been advocated as a treatment for propylene glycol overdose, given its mechanism of inhibiting alcohol dehydrogenase, which would limit lactic acid production.^4^ In cases of severe lactic acidosis unresponsive to pharmacologic therapy, renal dialysis is often required.

In our patient, the primary presenting symptom following an excess ingestion of both ethanol as well as propylene glycol was central nervous system depression. In this case, the patient was profoundly obtunded to a degree requiring intubation for airway protection. More research is needed to elucidate the effects of propylene glycol toxicity in the setting of acute alcohol toxicity, with potentially potentiating effects on central nervous system depression. We also observed a mild metabolic acidosis with elevated lactate levels, which resolved with conservative measures. Notably, patients with significant liver or renal disease would likely be at increased risk of severe metabolic acidosis if exposed to large quantities of propylene glycol. We conclude that propylene glycol toxicity may be of increasing clinical relevance and should be considered in patients with an elevated blood alcohol level and unexplained osmolal gap.
